# Influence of Socioeconomic Status on the Retail Food Environment in Alicante

**DOI:** 10.3390/nu16234127

**Published:** 2024-11-28

**Authors:** Iván Hernández-Caravaca, Alba Martínez-García, Eva María Trescastro-López, Ángel Plaza-Gavaldón, Julio Martí-Cremades, Joaquín Moncho

**Affiliations:** 1 Department of Community Nursing, Preventive Medicine and Public Health and History of Science, University of Alicante, 03690 Alicante, Spain; ivan.hernandez@ua.es (I.H.-C.); angelpg270@gmail.com (Á.P.-G.); marticremades21@gmail.com (J.M.-C.); joaquin.moncho@ua.es (J.M.); 2 Balmis Research Group in History of Science, Health Care and Food, University of Alicante, 03690 Alicante, Spain; eva.trescastro@ua.es; 3 Research Group on Applied Dietetics, Nutrition and Body Composition, University of Alicante, 03690 Alicante, Spain; 4 Department of Nursing, University of Alicante, 03690 Alicante, Spain; 5 Alicante Institute for Health and Biomedical Research (ISABIAL), Group 23, 03550 Alicante, Spain

**Keywords:** food environment, food availability, food access, NEMS-S-MED, socioeconomic status

## Abstract

Background/Objectives: Unhealthy dietary habits are associated with chronic non-communicable diseases and may contribute to increased mortality in all countries of the world. Food environments determine the accessibility, availability, and promotion of food, thus playing an important role in people’s diets, but they are context-dependent. The aim of this study was to evaluate the availability and accessibility of food in food outlets in different neighborhoods of the city of Alicante. Methods: Cross-sectional study. Trained researchers conducted food store audits using the validated Nutrition Environment Measures Survey in Stores for Mediterranean contexts (NEMS-S-MED) tool. Data were collected from food stores within a socioeconomically diverse sample in Alicante (*n* = 63). We compared the availability and affordability of “healthier–less healthy” food pairs, scores between food store types (supermarkets, specialized, convenience stores, and others), and socioeconomic levels in Alicante in 2022. Results: The present study highlights that the food stores with the greatest availability and accessibility to healthy foods are supermarkets, as opposed to specialized stores and convenience stores. In addition, differences were found in the accessibility and availability of healthy foods by neighborhoods with different income levels, being more accessible in the residential neighborhood with the highest income level. Tourism could explain the differences in the food retail environment, with a high-income neighborhood showing similar results to low-income neighborhoods. Conclusions: The results obtained invite reflection on the development and adoption of policy strategies that promote the availability and accessibility of healthy food in the most disadvantaged areas.

## 1. Introduction

Obesity and chronic non-communicable diseases (NCDs) have doubled both globally and in Spain in recent years [1,2,3]. This increase is concerning, as they are one of the leading causes of death in the world and represent genuine public health problems [4].

Unhealthy dietary habits are associated with these chronic pathologies and may contribute to increased mortality in all countries of the world [5]. For this reason, and due to the complexity of dietary behaviors and the wide range of influences that may be present in the diet of the population, improving diet requires the active collaboration of different elements involved in the food system [5].

The food environment determines the accessibility, availability, and promotion of food, thus playing an important role in people’s diets [6]. It can therefore influence a population’s food choices and encompass both foods consumed at home and foods obtained outside the home, including vending machines, takeaways, cafeterias, restaurants, supermarkets, and food stores [7]. There is also evidence of socioeconomic inequalities in access to food depending on the characteristics of the environment [8,9]. Hence, understanding food environments and how they differ between neighborhoods of different socioeconomic statuses can be a key part of tackling obesity and NCDs such as cancer, diabetes, or hypercholesterolemia. 

Previous research has assessed the relationship between the consumer food environment and the health status of the population [9,10,11,12], but it is context-dependent, and therefore, more studies are needed to determine how this environment influences each country and/or city [12,13]. Nonetheless, there is little information on the characteristics of the retail food environment in different southern European countries and, more specifically, in those with a Mediterranean context. Particularly, there are no studies assessing the food environment of cities in the Valencian Community (Spain), which is characterized by its Mediterranean setting.

For this reason, in order to extend the existing evidence and to fill the gap in the knowledge of the retail food environment of Mediterranean cities, the aim of this study was to evaluate the availability and accessibility of food in food outlets in different neighborhoods of the city of Alicante, taking into account the income level of the population and the types of food stores.

## 2. Materials and Methods

### 2.1. Study Design and Sample 

This is a cross-sectional descriptive observational study carried out in Alicante, a city on the Mediterranean coast of south-eastern Spain. According to the sources of the Spanish National Institute of Statistics (INE), Alicante is classified into 8 districts with different income levels [14].

In consideration of the mean income per capita and the mean household income, as reported by the National Institute of Statistics [14], four districts of Alicante exhibiting disparate income levels were selected. Of the selected districts, the lowest income brackets were identified in Districts 3 and 5, while Districts 1 and 4 exhibited the highest income levels. District 3 reported an average income per person of EUR 9803 and an average income per household of EUR 25,457; District 5 reported an average income per person of EUR 8655 and an average income per household of EUR 23,621. In contrast, District 1 exhibited an average income per person of EUR 1785 and an average income per household of EUR 38,709; District 4 had an average income per person of EUR 12,788 and an average income per household of EUR 34,235.

A neighborhood was selected within each district, with the objective of obtaining the most representative sample of the sociodemographic profile of the population. The selected neighborhoods were “Carolinas Altas” (District 3), “Virgen del Carmen” (District 5), “Centro” (District 1), and “Vistahermosa” (District 4).

### 2.2. Data Collection

Data collection was carried out in all food outlets in each neighborhood during business hours and on weekdays during the month of April 2022. The data were collected by two trained raters according to a protocol established for the completion of the survey. The following sales outlets were included: greengrocers, bakeries, butchers, and fishmongers, among others, or mixed sales outlets, such as convenience stores, hypermarkets, or supermarkets. Food markets and food galleries were excluded because standard tools for measuring healthy foods may not capture the effect of these retailers, following the procedure of previous studies [12,15].

The NEMS-S-MED survey [16] was used using mobile devices and the Google Forms platform. This survey, validated for Spain, assesses the availability and price of twelve food groups: fresh fruit, vegetables, nuts, beverages, bread, cereals and pastries, milk and dairy products, eggs, oil and butter, rice, pulses, and meat and fish [16].

### 2.3. Measures

The established NEMS-S-MED score [16] was used to analyze and evaluate the food establishments in the different districts. This score ranges from 0 to 49 points and takes into account both the availability of different food groups (0–37 points) and the price of food (0–12 points). The higher the score, the greater the availability and accessibility of healthy options in the establishment. The score was calculated for each food outlet in order to study differences by type of food store and neighborhood. 

### 2.4. Data Analysis

A descriptive analysis was carried out by calculating means and standard deviations for quantitative variables (food prices) and frequencies and percentages for qualitative variables (food availability and healthy and less healthy food) by type of establishment and neighborhood.

For analysis, food stores were classified into supermarkets (including discounters), convenience stores (including gas stations), and traditional/specialized establishments (greengrocers, butchers, fishmongers, and bakers), based on previous research [12,16,17]. 

In order to study the differences in the availability of the different foods included in this study according to the type of establishment and neighborhood, the Chi-square test or Fisher’s exact test was used. Furthermore, for the analysis, foods were categorized according to their content (higher or lower) of fat, sugar, and salt (FSS). The McNemar test and the Wilcoxon rank test for paired samples were used to compare the availability of foods with higher or lower FSS content, as well as the price of these foods in the different establishments, respectively. The Kruskal–Wallis test was used to compare food prices by neighborhood. Finally, linear mixed models were fitted with the dependent variable NEMS-S-MED total score and availability score in each case and fixed effects: neighborhood and type of establishment. In both models, the possible interaction effect between the independent variables was tested. Values were considered significantly different when *p* < 0.05. 

## 3. Results

### 3.1. Descriptive Data

Data were collected from 63 food stores. Of these, almost half of them were (49.3%, *n* = 31) specialized stores (butchers: 15.9%; bakeries: 15.9%; greengrocers 15.9%; and herbalists: 1.6%), followed by supermarkets (25.4%, *n* = 16) and convenience stores (25.4%, *n* = 16). The types of food stores by neighborhood and economic level are summarized in Table 1. No statistically significant differences were identified with regard to the types of food stores in each neighborhood (*p* = 0.545) or socioeconomic level (*p* = 0.140).

### 3.2. Food Availability and Price

Table 2 shows the availability of foods collected in the NEMS-S-MED survey by type of food store and by neighborhood. The products that showed an overall availability of more than 70% were eggs, cola, and commercial confectionery. Less common items were fresh fish (9.5%), processed and unprocessed frozen fish (19% and 20.6%, respectively), skimmed and sugar-free yogurts (19% and 22.2%, respectively), and whole rice (22.2%). Mean availability across all food items was 84.7% in supermarkets, 34.9% in convenience stores, and 24% in specialized stores. All the items studied (*n* = 38) showed statistically significant differences between the type of store and their availability (*p* < 0.05). However, the biggest differences between supermarkets and convenience stores were found in fresh fruit, fresh and frozen vegetables and nuts (88.8% average availability in supermarkets vs 18.8% in convenience stores), skimmed milk, skimmed yogurt and cheeses in general (92.2% vs. 26.6%), fresh meats in general (68.8% vs. 0%), and finally, in both unprocessed and processed frozen fish (68.8% vs. 6.3%).

When the availability of the items studied was compared among the different neighborhoods, statistically significant differences were found in fresh fruit, 100% juice, not-100% juice, whole bread, and low-sugar cereals, as well as in both unprocessed and processed frozen fish (*p* < 0.05). Differences were also observed for fresh vegetables, brown rice, and unsweetened/sweetener-free yogurts, which, although not significant at the usual level, showed a value of *p* < 0.07. The availability of all these items was higher in neighborhoods with higher socioeconomic status in almost all cases (Table 2).

Furthermore, the food items with more FSS showed a higher availability in products like salty nuts, not-100% juice, whole milk, white rice, regular cereals, and yogurt with sugar compared with the less FSS ones (*p* < 0.05). On the other hand, the majority (three out of four) of the prices that showed statistically significant differences were higher in the less FSS group (100% juice, olive oil, and whole rice) (Table 3).

When the prices were compared between the different neighborhoods, statistically significant differences were found in the price of the chicken (*p* = 0.045), as well as a trend in the price of semi-skimmed milk, showing high-level economic neighborhoods having the highest prices. For the rest of the food items, no differences were found (*p* > 0.05) (Supplementary Material Table S1). 

### 3.3. NEMS-S-MED Score

**Figure 1:**
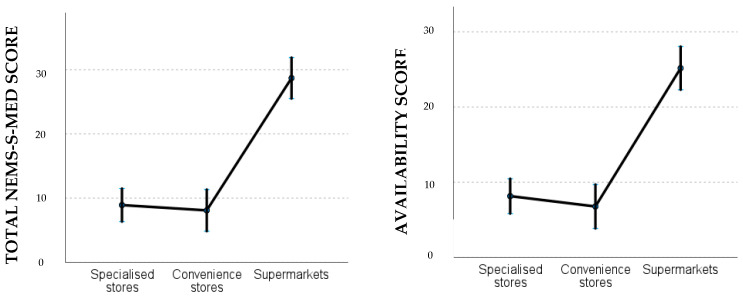
Total and availability scores from the NEMS-S-MED survey by type of food store.

**Figure 2:**
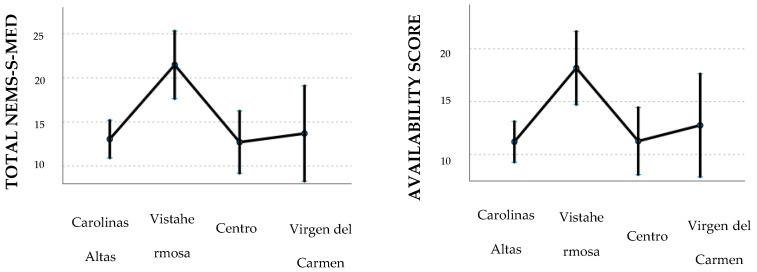
Total and availability scores from the NEMS-S-MED survey by neighborhood.

Table 4 and Figure 1 and Figure 2 illustrate the outcomes of the linear mixed models, which were fitted with the total NEMS-S-MED score and the availability score as dependent variables. 

The estimated marginal means (Figure 1) showed that supermarkets presented a higher total score (mean = 28.703; 95%CI: 25.511, 31.895) and availability score (mean = 25.163; 95%CI: 22.288, 28.038), followed by specialized stores (mean total score: 8.911; 95%CI: 6.332, 11.490; mean availability score: 8.138; 95%CI: 5.815, 10.461) and finally convenience stores (mean total score: 8.088; 95%CI: 4.841, 11.334; mean availability score: 6.762; 95%CI: 3.838, 9.686). In terms of scores by neighborhood (Figure 2), the high-income residential neighborhood “Vistahermosa” has the highest total and availability scores (mean total score: 21.471; 95%CI: 17.635, 25.306; mean availability score: 18.182; 95%CI: 14.727, 21.636), followed by the other neighborhoods with similar average scores (mean total score “Centro”: 12.713; “Carolinas altas”: 13.058; and “Virgen del Carmen”: 13.694).

The results of the linear mixed models demonstrate a statistically significant impact of food store type (*p* < 0.01) and neighborhood (*p* < 0.01) on both the total and availability scores of the NEMS-S-MED. The total NEMS-S-MED score for convenience and specialized stores was approximately 20 points lower than that for supermarkets (−20.6 and −19.8, respectively, *p* < 0.01), while the availability score was between 18.4 and 17 points lower (*p* < 0.01). No significant differences were observed in the scores obtained from convenience and specialized stores. The adjusted effect for the “Vistahermosa” neighborhood (characterized by high levels of wealth) was approximately 7.8 points higher than that observed in the “Virgen del Carmen” neighborhood (characterized by low levels of wealth) and approximately 7 points higher than in the other neighborhoods (*p* < 0.05). The neighborhoods of “Carolinas Altas” and “Centro” presented scores similar to those of “Virgen del Carmen”.

## 4. Discussion

This study evaluates the food retail food environment in Alicante (Spain) as an example of a Mediterranean city in southwestern Europe. 

The present study demonstrates that supermarkets are the food stores with the greatest availability and accessibility to healthy foods, in contrast to specialized stores and convenience stores. Previous research carried out in different countries (Malta, China, UK, Germany, US, and Australia), as well as in Spain, has obtained similar results [12,18,19,20,21,22,23]. This is due to the fact that supermarkets generally offer a wider range of foods than single-product stores. However, as shown in the previous section, there is also greater availability of unhealthy and ultra-processed foods. Earlier research highlights the same issue, underlining that this offer can have an opposite or detrimental effect by offering both healthy and unhealthy foods [12,24]. 

On the other hand, specialized stores (butchers, fishmongers, greengrocers, etc.) offer fresh and healthy food and generally do not have a wide range of unhealthy food, as they sell the products they specialize in. Despite this, they have similar scores as convenience stores and lower scores than supermarkets. Therefore, encouraging people to shop in these types of establishments could be more useful in encouraging people to eat a healthier diet. Nevertheless, this strategy may be difficult to implement, as previous national studies have found an increase in supermarket shopping to the detriment of traditional small establishments [25,26]. Similarly, research has shown that due to the food transition and the “Westernization” of Mediterranean food environments, the number of supermarkets and hypermarkets has been increasing over the last decades [17,27,28]. Furthermore, marketing strategies are more prevalent in supermarkets than in specialized food stores and are primarily designed to boost the sale of unhealthy foods [25,28], which may lead to an increase in unhealthy product consumption.

Concerning convenience stores, according to our results, they offer more unhealthy products than other establishments and were the lowest rated according to the NEMS-S-MED survey. These results are in line with studies carried out in the USA and the UK [29,30], where fewer healthy products are available in convenience stores compared with supermarkets. Similarly, previous studies in Madrid and Barcelona show a similar trend [12,31].

In terms of the availability and price of products with higher or lower FSS content, in our study, it was observed that products with a less healthy profile, such as white rice, sweetened yogurts, or sugar-containing cereals, were more readily available and offered at a lower price point than wholegrain rice, sugar-free yogurt, and cereals. A similar trend is observed in studies carried out in Madrid [12], where it was found that most healthier foods (such as 100% juice versus not-100% juice, low-sugar cereals versus regular cereals, or whole rice versus white rice) were less available than their less healthy counterparts, suggesting that households with limited financial resources face greater challenges in accessing healthy foods. Previous research [32,33] has shown that this can directly influence food purchasing decisions between different socioeconomic levels, creating an economic barrier for low-income people who tend to choose less healthy options because of the price difference. This underscores the importance of considering these access and equity issues in healthy eating when formulating food policies and strategies. In this regard, food price policies are recommended as one of the most potentially effective public health measures to promote healthy diets [34].

Apart from the aforementioned, in the present study, it was observed that in neighborhoods with a high income level, the score was higher than in those with a lower income level. A similar effect has been observed in previous research in Malta [18], the UK [19], Australia [20], and Brazil [35], as well as in other cities [12,23]. Nevertheless, there is considerable variability in this aspect, which underscores the need for studies in different contexts to confirm existing results in different countries. It is also noteworthy that, despite comparable income levels, significant discrepancies in scores were observed between two neighborhoods with similar (high) income levels in our study. In this regard, it is pertinent to highlight the distinctive characteristics of the “Centro” neighborhood. Despite the high income level of the residents, the retailers in this area demonstrate performance outcomes that are comparable to those observed in low-income neighborhoods. This may be attributed to the fact that the “Centro” neighborhood experiences the highest influx of tourists in the city of Alicante, attracted by the cultural and leisure offerings, as well as the proximity to the beach, which characterizes this neighborhood. This phenomenon appears to be influencing the food supply in the area undergoing a process of “Westernization”, with results differing from those observed in other high-income neighborhoods (Vistahermosa). Consequently, future research should examine the impact of tourism on the food supply in greater depth in other areas worldwide.

Research in different contexts that increases knowledge about the food retail environment can help to develop effective policies on a global scale that improve the situation and access to healthy food for the population, regardless of where they live. In Spain, in particular, there is a need for such food policies that act on the environment and not only at the level of individual education. For this reason, studies in different national contexts are important to know what is happening in each area.

In light of the above, it would therefore be interesting to promote the availability and accessibility of healthy food in the most disadvantaged areas through policy strategies that can enhance people’s ability to purchase better quality products, considering their economic, geographic, and cultural circumstances.

### Limitations and Strengths

With regard to the limitations of this study, it should be noted that although the sample does not represent a large number, all the establishments present in the selected neighborhoods have been included. A larger sample would strengthen the findings and allow for more nuanced conclusions. On the other hand, a convenience sample was used in order to ensure socioeconomic diversity, so extrapolation of the results should be performed cautiously. In addition, this paper lacks direct data on consumer purchases or intake or the presence of in-store marketing campaigns. Future research, therefore, could be carried out taking into account the aspects mentioned above.

Notwithstanding, the present study has important strengths. First and foremost, it is the first study carried out in a medium-sized city such as Alicante. This study considers that the food supply in outlets is influenced not only by the economic level of the neighborhood but also by the offerings to tourists. In addition, a tool validated in the Mediterranean context has been used to describe and compare the availability and accessibility of food in the retail food environment and, at the same time, to compare it with previous studies carried out in other Spanish cities.

## 5. Conclusions

This study, carried out in the city of Alicante, shows that supermarkets have greater availability and accessibility to healthy foods than specialized establishments and convenience stores. In addition, differences were found in the accessibility and availability of healthy foods by neighborhoods with different income levels, being more accessible in the residential neighborhood with the highest income level. 

The results suggest that tourism could influence the retail food environment, with high-income neighborhoods exhibiting comparable outcomes to those observed in low-income areas. Future research should investigate this aspect further in order to corroborate it. 

The results obtained encourage reflection on the development and implementation of policy strategies that facilitate the availability and accessibility of healthy food in the most disadvantaged areas.

## Figures and Tables

**Table 1:** Percentage of types of food stores in each neighborhood and by socioeconomic level.

Type of Food Store	Total		Neighborhood							
			High Economic Level				Low Economic Level			
			Vistahermosa		Centro		Carolinas Altas		Virgen del Carmen	
	*n*	%	*n*	%	*n*	%	*n*	%	*n*	%
Supermarkets	16	25.4	4	40.0	5	41.7	6	16.7	1	20.0
Convenience stores	16	25.4	2	20.0	2	16.7	10	27.8	2	40.0
Specialized stores	31	49.3	4	40.0	5	41.7	20	55.6	2	40.0
Total	63	100	10	100	12	100	36		5	100

**Table 2:** Availability of the food items included in NEMS-S-MED by type of food store and neighborhood.

Food Item	Type of Food Store					Neighborhood					
	Supermarket	Convenience Stores	Specialized Stores	Total	Sig	Vistahermosa	Centro	Carolinas Altas	Virgen del Carmen	Total	Sig ^(b)^
Fresh fruit	93.8%	18.8%	35.5%	46.0%	<0.001 ^(a)^	70.0%	66.7%	30.6%	60%	46.0%	0.037
Fresh vegetables	93.8%	18.8%	38.7%	47.6%	<0.001 ^(a)^	70.0%	66.7%	33.3%	60%	47.6%	0.067
Potatoes	93.8%	12.5%	41.9%	47.6%	<0.001 ^(a)^	60.0%	66.7%	36.1%	60%	47.6%	0.204
Frozen vegetables	75.0%	18.8%	9.7%	28.6%	<0.001 ^(b)^	60.0%	33.3%	19.4%	20%	28.6%	0.093
Unprocessed nuts	87.5%	25.0%	32.3%	44.4%	<0.001 ^(a)^	80.0%	33.3%	41.7%	20%	44.4%	0.079
Salty nuts	81.3%	93.8%	29.0%	58.7%	<0.001 ^(a)^	80.0%	41.7%	58.3%	60%	58.7%	0.353
Light cola drink	93.8%	93.8%	54.8%	74.6%	0.002 ^(b)^	70.0%	66.7%	80.6%	60%	74.6%	0.553
Regular cola drink	93.8%	93.8%	61.3%	77.8%	0.012 ^(b)^	70.0%	66.7%	86.1%	60%	77.8%	0.236
Juice 100%	68.8%	12.5%	9.7%	25.4%	<0.001 ^(b)^	50.0%	41.7%	11.1%	40%	25.4%	0.012
Not-100% juice	87.5%	75.0%	48.4%	65.1%	0.018 ^(a)^	70.0%	33.3%	77.8%	40%	65.1%	0.041
Whole bread	81.3%	25.0%	35.5%	44.4%	0.002 ^(a)^	90.0%	50.0%	33.3%	20%	44.4%	0.006
Low sugar cereals	56.3%	--	19.4%	23.8%	<0.001 ^(b)^	30.0%	41.7%	19.4%	0%	23.8%	0.270
Regular cereals	93.8%	37.5%	9.7%	38.1%	<0.001 ^(a)^	60.0%	50.0%	30.6%	20%	38.1%	0.248
Confectionery	93.8%	100.0%	54.8%	76.2%	<0.001 ^(b)^	80.0%	58.3%	80.6%	80%	76.2%	0.453
Skimmed milk	100.0%	37.5%	22.6%	46.0%	<0.001 ^(a)^	70.0%	58.3%	38.9%	20%	46.0%	0.185
Semi-skimmed milk	100.0%	56.3%	25.8%	52.4%	<0.001 ^(a)^	70.0%	66.7%	47.2%	20%	52.4%	0.210
Whole milk	100.0%	75.0%	29.0%	58.7%	<0.001 ^(a)^	80.0%	66.7%	50.0%	60%	58.7%	0.359
Skimmed yogurt	68.8%	--	3.2%	19.0%	<0.001 ^(b)^	30.0%	33.3%	13.9%	0%	19.0%	0.277
Semi-hard cheese	100.0%	37.5%	19.4%	44.4%	<0.001 ^(a)^	70.0%	50.0%	33.3%	60%	44.4%	0.156
Fresh cheese	100.0%	31.3%	22.6%	44.4%	<0.001 ^(a)^	70.0%	41.7%	36.1%	60%	44.4%	0.255
Eggs	100.0%	62.5%	77.4%	79.4%	0.018 ^(b)^	100.0%	75.0%	72.2%	100%	79.4%	0.181
Olive oil	93.8%	43.8%	25.8%	47.6%	<0.001 ^(a)^	80.0%	41.7%	38.9%	60%	47.6%	0.124
Sunflower oil	75.0%	37.5%	19.4%	38.1%	<0.001 ^(a)^	40.0%	41.7%	33.3%	60%	38.1%	0.639
Salt-free butter	87.5%	12.5%	9.7%	30.2%	<0.001 ^(b)^	40.0%	41.7%	25.0%	20%	30.2%	0.605
Regular butter	93.8%	50.0%	12.9%	42.9%	<0.001 ^(a)^	50.0%	50.0%	33.3%	80%	42.9%	0.210
Whole rice	75.0%	6.3%	3.2%	22.2%	<0.001 ^(b)^	40.0%	33.3%	11.1%	40%	22.2%	0.054
White rice	100.0%	75.0%	29.0%	58.7%	<0.001 ^(a)^	70.0%	50.0%	58.3%	60%	58.7%	0.854
Legumes	100.0%	75.0%	41.9%	65.1%	<0.001 ^(a)^	90.0%	75.0%	55.6%	60%	65.1%	0.182
Beef	62.5%	--	29.0%	30.2%	<0.001 ^(b)^	50.0%	16.7%	27.8%	40%	30.2%	0.345
Chicken	75.0%	--	29.0%	33.3%	<0.001 ^(a)^	50.0%	25.0%	30.6%	40%	33.3%	0.628
Sausage	93.8%	56.3%	29.0%	52.4%	<0.001 ^(a)^	70.0%	58.3%	41.7%	80%	52.4%	0.225
Fresh fish	37.5%	--	--	9.5%	<0.001 ^(b)^	20.0%	8.3%	8.3%	0%	9.5%	0.640
Unprocessed frozen fish	68.8%	6.3%	--	19.0%	<0.001 ^(b)^	50%	25.0%	8.3%	20%	19%	0.018
Processed frozen fish	68.8%	6.3%	3.2%	20.6%	<0.001 ^(b)^	50%	25.0%	11.1%	20%	20.6%	0.045
Canned tuna	87.5%	62.5%	29.0%	52.4%	<0.001 ^(a)^	80%	50.0%	44.4%	60%	52.4%	0.242
Plant-based beverage with added sugar	87.5%	18.8%	3.2%	28.6%	<0.001 ^(b)^	20%	41.7%	60%	16.7%	28.6%	0.030
Plant-based beverage without added sugar	75.0%	12.5%	3.2%	23.8%	<0.001 ^(b)^	20%	33.3%	50%	13.9%	23.8%	0.074
Yogurts without sugar/sweetener	81.3%	0%	3.2%	22.2%	<0.001 ^(b)^	40%	41.7%	13.9%	0%	22.2%	0.061
Yogurts with sugar	87.5%	31.3%	16.1%	38.1%	<0.001 ^(a)^	60%	33.3%	36.1%	20%	38.1%	0.461

^(a)^ Chisquare-test; ^(b)^ Fisher exact test.

**Table 3:** Comparison of availability and price of food items divided by its composition in more or less content of saturated fat, salt, and sugar.

	Availability				Price			
	Less FSS ^(1)^	High FSS ^(1)^	Difference	*p*-Value ^(2)^	Less FSS ^(1)^	High FSS ^(1)^	Difference	*p*-Value ^(2)^
Nuts (unprocessed vs. salty)	44.44%	58.73%	−14.29%	0.049	-	-	-	-
Juice (100% vs. not 100%)	25.40%	65.08%	−39.68%	<0.001	2.32 (1.34)	1.92 (0.94)	0.40	0.037
Cola (light vs. regular)	74.60%	77.8%	−3.17%	0.500	2.06 (0.87)	2.21 (0.99)	−0.15	0.317
Milk (Skimmed vs. whole)	46.03%	58.73%	−12.70%	0.021	1 (0.00)	1.14 (0.55)	−0.14	1.000
Cheese (Fresh vs. semi-hard)	44.44%	44.44%	0.00%	1	-	-	-	-
Oil (olive vs. sunflower)	47.62%	38.10%	9.52%	0.146	5.36 (1.36)	3.75 (1.59)	1.61	0.000
Rice (whole vs. white)	22.22%	58.73%	−36.51%	<0.001	2.11 (1.23)	1.51 (0.77)	0.60	0.004
Meat (chicken vs. beef)	33.33%	30.16%	3.17%	0.5	6.60 (1.74)	12.76 (2.73)	−6.16	0.000
Frozen fish (unprocessed vs. processed)	19.05%	20.63%	−1.59%	1	-	-	-	
Cereals (Low sugar vs. regular)	15.87%	38.10%	−22.22%	0.001	-	-	-	
Yogurts (without sugar/sweetener vs. with sugar)	22.22%	38.10%	−15.87%	0.006	3.60 (3.66)	2.95 (1.91)	0.65	0.092
Plant-based beverage (without added sugar vs. with added sugar)	23.81%	28.57%	−4.76%	0.25	1.62 (0.53)	1.41 (0.54)	0.21	0.209

^(1)^ FSS (saturated fat, salt, and sugar); ^(2)^ McNemar test.

**Table 4:** Linear mixed model for the NEMS-S-MED total and availability scores.

Parameter	NEMS-S-MED Total		NEMS-S-MED Availability	
Fixed Effects	Estimate	95%CI	Estimate	95%CI
Type of food retailer ^(a)^				
Supermarket (Base)				
Convenience store	−20.615 ^(a)^	−24.996, −16.235	−18.401 ^(a)^	−22.347, −14.455
Specialized stores	−19.792 ^(a)^	−23.608, −15.976	−17.025 ^(a)^	−20.462, −13.588
Neighborhood by economic level ^(a)^				
Low—Virgen del Carmen (Base)				
Low—Carolinas altas	−0.635	−6.401, 5.130	−1.556	−6.750, 3.637
High—Vistahermosa	7.777 ^(b)^	1.128, 14.426	5.420	−0.569, 11.409
High—Centro	−0.980	−7.461, 5.500	−1.493	−7.330, 4.344

^(a)^ *p* < 0.01 ^(b)^ *p* < 0.05.

## Data Availability

The datasets used and/or analyzed during the current study are available from the corresponding author. The data are not publicly available due to it has been collected in this study and there is no link or website to redirect to.

## Supplementary Materials

The following supporting information can be downloaded at https://www.mdpi.com/article/10.3390/nu16234127/s1, Table S1: Comparison of food prices by neighborhood.
