# Supplementary material for: Influence of Socioeconomic Status on the Retail Food Environment in Alicante
Source: Nutrients. 2024 Nov 28;16(23):4127. doi: 10.3390/nu16234127 (PMC11644795; doi:10.3390/nu16234127)
Supplement: Supplementary file 1 [file nutrients-16-04127-s001.zip › nutrients-3343936-supplementary.pdf]

SUPPLEMENTARY MATERIAL

Table S1. Comparison of food prices by neighbourhood.

Price	Neighborhoods	N	Mean	SD*	Min	Max	Sig
Apple (€/kg)	Vistahermosa	7	2.44	0.34	1.99	3.05	0.077
	Centro	8	1.85	0.74	0.99	3.22	
	Carolinas altas	11	2.44	0.68	0.99	2.99	
	Virgen del Carmen	3	1.60	0.53	0.99	2.00	
	Total	29	2.19	0.68	0.99	3.22	
Tomato (€/kg)	Vistahermosa	7	2.72	0.96	1.50	4.00	0.153
	Centro	8	2.74	1.22	1.59	5.25	
	Carolinas altas	10	1.67	0.52	0.95	2.39	
	Virgen del Carmen	3	2.50	1,.0	1.70	4.00	
	Total	28	2.33	1.03	0.95	525	
Frozen spinach (€/kg)	Vistahermosa	4	3.93	0.70	2.90	4.45	0.270
	Centro	4	2.64	2.25	1.22	5.96	
	Carolinas altas	3	5.22	3.03	1.80	7.60	
	Virgen del Carmen	1	1.75	-	1.75	1.75	
	Total	12	3.64	2.14	1.22	7.60	
Light cola drink (€/l)	Vistahermosa	7	2.28	1.09	0.89	4.00	0.386
	Centro	8	1.58	0.74	0.75	3.00	
	Carolinas altas	29	2.18	0.81	0.90	3.80	
	Virgen del Carmen	3	1.65	1.17	0.95	3.00	
	Total	47	2.06	0.87	0.75	4.00	
Regular cola drink (€/l)	Vistahermosa	2	1.95	1.49	0.90	3.00	0.887
	Centro	2	2.50	0.71	2.00	3.00	
	Carolinas altas	8	2.25	1.04	1.00	3.00	
	Virgen del Carmen	2	2.00	1.41	1.00	3.00	
	Total	14	2.21	0.99	0.90	3.00	
Juice 100% (€/l)	Vistahermosa	5	2.59	1.30	1.72	4.85	0.438
	Centro	5	1.59	1.18	0.70	3.49	
	Carolinas altas	4	2.70	1.56	1.35	4.92	
	Virgen del Carmen	2	2.75	1.77	1.50	4.00	
	Total	16	2.32	1.34	0.70	4.92	
Not juice 100% (€/l)	Vistahermosa	7	1.81	0.77	0.66	2.75	0.144
	Centro	4	1.15	0.60	0.65	2.00	
	Carolinas altas	28	2.11	0.98	0.83	4.00	
	Virgen del Carmen	2	1.25	0.07	1.20	1.30	
	Total	41	1.92	0.94	0.65	4.00	

Low sugar cerals (€/kg)	Vistahermosa	2	4.08	3.01	1.95	6.20	0.232
	Centro	4	4.59	1.88	2.10	6.46	
	Carolinas altas	4	7.97	2.58	5.57	10.80	
	Virgen del Carmen	0	-	-	-	-	
	Total	10	5.84	2.79	1.95	10.80	
Regular cereals (€/kg)	Vistahermosa	6	5.23	1.77	1.98	6.90	0.353
	Centro	6	5.93	1.92	2.40	7.77	
	Carolinas altas	11	4.81	1.46	3.10	7.69	
	Virgen del Carmen	1	6.40	-	6.40	6.40	
	Total	24	5.26	1.64	1.98	7.77	
Skimmed milk (€/l)	Vistahermosa	0	-	-	-	-	1.000
	Centro	1	1.00	-	1.00	1.00	
	Carolinas altas	2	1.00	0.00	1.00	1.00	
	Virgen del Carmen	0	-	-	-	-	
	Total	3	1.00	0.00	1.00	1.00	
Semi-skimmed milk (€/l)	Vistahermosa	7	1.01	0.15	0.85	1.30	0.061
	Centro	8	1.32	1.38	0.60	4.70	
	Carolinas altas	17	1.15	0.20	0.95	1.75	
	Virgen del Carmen	1	0.68	-	0.68	0.68	
	Total	33	1.14	0.68	0.60	4.70	
Whole milk (€/l)	Vistahermosa	8	1.09	0.37	0.76	1.80	0.084
	Centro	8	1.23	1.13	0.60	3.98	
	Carolinas altas	18	1.16	0.19	0.99	1.75	
	Virgen del Carmen	3	0.90	0.18	0.69	1.00	
	Total	37	1.14	0.55	0.60	3.98	
Olive oil (€/l)	Vistahermosa	8	5.02	1.12	3.99	7.00	0.181
	Centro	5	5.19	1.47	3.95	7.72	
	Carolinas altas	14	5.83	1.39	4.30	9.26	
	Virgen del Carmen	3	4.36	1.42	3.49	6.00	
	Total	30	5.36	1.36	3.49	9.26	
Sunflower oil (€/l)	Vistahermosa	4	3.24	0.25	2.99	3.59	0.345
	Centro	5	3.57	1.32	2.40	5.84	
	Carolinas altas	12	4.15	2.03	2.50	10.40	
	Virgen del Carmen	3	3.14	0.89	2.13	3.79	
	Total	24	3.75	1.59	2.13	10.40	
Whole rice (€/kg)	Vistahermosa	4	1.62	0.19	1.45	1.85	0.366
	Centro	4	2.59	1.91	1.60	5.45	
	Carolinas altas	4	2.47	1.30	1.60	4.39	
	Virgen del Carmen	2	1.43	0.32	1.20	1.65	
	Total	14	2.11	1.23	1.20	5.45	

White rice (€/kg)	Vistahermosa	7	1.53	0.45	0.99	2.25	0.166
	Centro	6	1.79	1.81	0.68	5.44	
	Carolinas altas	21	1.49	0.36	0.95	1.99	
	Virgen del Carmen	3	1.05	0.13	0.95	1.20	
	Total	37	1.51	0.77	0.68	5.44	
Beef (€/kg)	Vistahermosa	5	13.29	2.04	11.69	16.80	0.949
	Centro	2	13.95	2.76	11.99	15.90	
	Carolinas altas	10	12.22	3.35	7.20	17.99	
	Virgen del Carmen	2	12.95	1.34	12.00	13.90	
	Total	19	12.76	2.73	7.20	17.99	
Chicken (€/kg)	Vistahermosa	5	7.07	2.02	4.20	9.70	0.045
	Centro	3	8.33	0.97	7.50	9.40	
	Carolinas altas	11	5.77	1.55	3.50	7.96	
	Virgen del Carmen	2	7.34	0.06	7.30	7.38	
	Total	21	6.60	1.74	3.50	9.70	
Fresh fish (hake) (€/kg)	Vistahermosa	2	15.97	1.44	14.95	16.99	0.304
	Centro	1	20.40	-	20.40	20.40	
	Carolinas altas	3	14.38	3.60	11.30	18.33	
	Virgen del Carmen	0	-	-	-	-	
	Total	6	15.91	3.32	11.30	20.40	
Plant-based beverage with added sugar (€/l)	Vistahermosa	6	1.41	0.39	0.85	1.99	0.445
	Centro	5	1.34	0.76	0.70	2.51	
	Carolinas altas	6	1.58	0.53	1.02	2.50	
	Virgen del Carmen	1	0.85	-	0.85	0.85	
	Total	18	1.41	0.54	0.70	2.51	
Plant-based beverage without added sugar (€/l)	Vistahermosa	5	1.90	0.42	1.29	2.45	0.161
	Centro	4	1.28	0.52	0.80	1.98	
	Carolinas altas	5	1.76	0.47	1.40	2.50	
	Virgen del Carmen	1	0.85	-	085	0.85	
	Total	15	1.62	0.53	0.80	2,50	
Skimmed yogurts (€/kg)	Vistahermosa	4	2.39	0.68	1.75	3.30	0.990
	Centro	5	3.53	2.86	1.30	8.38	
	Carolinas altas	5	3.78	3.53	1.50	10.00	
	Virgen del Carmen	0	-	-	-	-	
	Total	14	3.30	2.61	1.30	10.00	
Yogurts without sugar/sweetener (€/kg)	Vistahermosa	3	1.89	1.24	0.98	3.30	0.276
	Centro	4	2.43	0.92	1.60	3.45	
	Carolinas altas	3	6.67	6.53	1.50	14.00	
	Virgen del Carmen	1	4.18	-	4.18	4.18	
	Total	11	3.60	3.66	0.98	14.00	

Yogurts with sugar (€/kg)	Vistahermosa	6	2.86	1.74	1.15	6.20	0.430
	Centro	4	1.91	1.05	1.20	3.45	
	Carolinas altas	13	3.34	2.22	1.68	10.00	
	Virgen del Carmen	1	2.58	-	2.58	2.58	
	Total	24	2.95	1.91	1.15	10.00	

* Standard deviation
